# Bone marrow aspirate concentrate quality is affected by age and harvest site

**DOI:** 10.1007/s00167-022-07153-6

**Published:** 2022-09-26

**Authors:** Carola Cavallo, Angelo Boffa, Laura de Girolamo, Giulia Merli, Elizaveta Kon, Luca Cattini, Emma Santo, Brunella Grigolo, Giuseppe Filardo

**Affiliations:** 1 Laboratorio RAMSES, IRCCS Istituto Ortopedico Rizzoli, Bologna, Italy; 2 Applied and Translational Research (ATR) Center, IRCCS Istituto Ortopedico Rizzoli, Bologna, Italy; 3 Laboratorio di Biotecnologie Applicate all’Ortopedia, IRCCS Istituto Ortopedico Galeazzi, Milan, Italy; 4 Humanitas Clinical and Research Center – IRCCS, Rozzano, Italy; 5 Humanitas University, Department of Biomedical Sciences, Pieve Emanuele, Italy; 6 Laboratorio di Immunoreumatologia e Rigenerazione Tissutale, IRCCS Istituto Ortopedico Rizzoli, Bologna, Italy

**Keywords:** Bone marrow aspirate concentrate, BMAC, MSCs, Iliac crest, Tibia, Harvest site, Age

## Abstract

**Purpose:**

To compare the number and properties of bone marrow stromal cells (BMSCs) collected from bone marrow aspirate concentrate (BMAC) obtained from different harvest sites and from patients of different ages.

**Methods:**

BMAC was obtained from two groups of patients based on age (*n* = 10 per group): 19.0 ± 2.7 years for the younger and 56.8 ± 12.5 for the older group. In the latter, BMAC was obtained from both iliac crest and proximal tibia for a donor-matched analysis. Mononucleated cell count and CFU-F assay were performed, together with phenotype characterization of BMSCs from iliac crest and proximal tibia, the study of chondrogenic and osteogenic differentiation capacity, histological staining and spectrophotometric quantification, and the analysis of mRNAs expression.

**Results:**

Cells derived from iliac crest and proximal tibia showed the same phenotypic pattern at flow cytometry, as well as similar chondrogenic and osteogenic potential. However, a significantly higher number of mononuclear cells per ml was observed in younger patients (3.8 ± 1.8 × 10^7^) compared to older patients (1.2 ± 0.8 × 10^7^) (*p* < 0.0005). The latter yield, obtained from the iliac crest, was significantly higher than resulting from the BMAC harvested from the proximal tibia in the same group of patients (0.3 ± 0.2 × 10^7^, *p* < 0.0005). This result was confirmed by the CFU-F analysis at day 10 (15.9 ± 19.4 vs 0.6 ± 1.0, *p* = 0.001) and day-20 (21.7 ± 23.0 vs 2.9 ± 4.2, *p* = 0.006).

**Conclusion:**

Harvest site and age can affect the quality of BMAC. BMSCs obtained from iliac crest and proximal tibia present comparable mesenchymal markers expression as well as osteogenic and chondrogenic differentiation potential, but iliac crest BMAC presents a four times higher number of mononucleated cells with significantly higher clonogenic capacity compared to the tibia. BMAC of younger patients also had a three-time higher number of mononucleated cells. The identification of BMAC characteristics could help to optimize its preparation and to identify the most suitable indications for this orthobiologic treatment in the clinical practice.

## Introduction

The use of mesenchymal stromal cells (MSCs) is gaining interest for musculoskeletal diseases [3]. In the traditional view, MSCs are multipotent cells with the potential to self-renew and differentiate into several different lineages including osteogenic, chondrogenic, adipogenic, and myogenic cell lines [20]. More recently, MSCs have been demonstrated to release several bioactive molecules with immunosuppressive and anti-inflammatory effects [23, 43], suggesting a key role in the response to tissue injuries not just by differentiating themselves, but also by inducing repair/regeneration processes at the injury site through the restoration of a pro-regenerative environment [6, 7]. Several sources of adult MSCs have been reported including bone marrow, synovial membranes, adipose tissue, tendons, skeletal muscle, and periosteum [60]. Despite the promising preclinical studies, regulatory concerns related to the use of expanded MSCs have limited their application in the clinical practice [28], fostering the search for alternative strategies to exploit the potential of MSC through a minimal manipulation approach. This allowed the emergence of cell-based products, prepared at the point of care (POC), capable of bypassing the strict regulations and issues related to cell manipulation and expansion [28]. Among them, bone marrow aspirate concentrate (BMAC) is one of the most studied and utilized [19, 24].

BMAC is a product that contains a combination of cells, platelets, and bioactive proteins such as growth factors and cytokines [25]. In particular, bone marrow MSCs (BMSCs) with their regenerative as well as anti-inflammatory and immunomodulatory properties are supposed to contribute to BMAC therapeutic potential [23, 55]. BMAC is prepared at the POC in a single sitting procedure consisting of bone marrow centrifugation leading to the separation of a product enriched in mononucleated cells, among which also BMSCs, ready to use/inject. Given its easy preparation, BMAC has been widely used in the clinical practice, demonstrating safety and positive results in the treatment of several musculoskeletal conditions [4, 9]. However, the real clinical potential of BMAC remains controversial and many aspects remain to be clarified to standardize and optimize this orthobiologic treatment. Among these factors, the harvest site and the age of the patient could play an important role in determining both the amount of contained BMSCs and their quality, and consequently the biological potential of BMAC.

Thus, the purpose of this study was to shed some light on the characteristics of BMSCs within BMAC based on harvest site and patient age, two main factors that may influence the biologic potential of BMAC for the treatment of musculoskeletal diseases. The hypothesis is that BMAC samples obtained from older patients contain a lower number of BMSCs compared to younger patients, while in the same patients BMAC obtained from the iliac crest contain more BMSCs compared to the proximal tibia.

## Material and methods

Patients scheduled to undergo an elective orthopedic procedure with BMAC treatment were enrolled. All the patients signed an informed consent before the procedure and Ethics Committee approval was obtained from the Rizzoli Orthopedic Institute, Bologna, Italy. The included patients have been divided into two groups based on age at the time of surgery (*n* = 10 per group). The mean age was 19.0 ± 2.7 years for the younger group and 56.8 ± 12.5 for the older group (patients with mild knee OA). Out of the 20 patients, only 2 were female, 1 for each group. In the younger group, bone marrow was collected via percutaneous aspiration from the anterior iliac crest. In the older group, percutaneous aspiration was performed to obtain bone marrow from both the anterior iliac crest and the proximal tibia. After processing through centrifugation, a small fraction of residual BMAC was sent to the laboratory of the same institute for the in vitro characterization.

### Mononucleated cell count and CFU-F assay

BMAC obtained from iliac crest and proximal tibia was diluted 1:4 in alpha minimum essential media (α-MEM, Gibco BRL, Rockville, MD, USA.) containing 15% of fetal bovine serum (FBS, Euroclone S.p.A., Milan, Italy), and the nucleated cells were counted with Turk dye (CARLO ERBA Reagents S.r.l, Milan, Italy). The clonogenic ability of cells derived from iliac crest and proximal tibia was assessed through the fibroblast colony-forming units (CFU-F) assay. Briefly, 5 × 10^5^ mononuclear cells were plated in a Petri dish and cultured for 10 and 20 days in α-MEM with 15% FBS. At 10 and 20 days, cells were fixed in methanol and stained with a 2% Crystal Violet Solution (Sigma-Aldrich, St. Louis, MO, USA). A cell aggregate containing more than 50 cells was identified as a colony originating from one clonal cell and counted for the purpose of this study.

### Phenotype characterization of BMSCs from iliac crest and tibia

BMAC obtained from iliac crest and proximal tibia was seeded into culture flasks (20 × 10^3^ mononuclear cells/cm^2^) and expanded for one passage in α-MEM with 15% FBS to select BMSCs. At confluence, BMSCs were detached and their immunophenotype was analyzed using specific mesenchymal and hematopoietic markers; 1 × 10^5^ cells were resuspended in phosphate-buffered saline (PBS) supplemented with 0.2% sodium azide (Sigma–Aldrich) and 2% FBS and incubated for 30 min at 4 °C with the following labeled mouse anti-human antibodies: CD73, CD105, CD106, CD31, CD34 (BD Pharmingen, Franklin Lakes, NJ, USA), CD90 (Biolegend, San Diego, CA, USA), and CD45 (DAKO, Santa Clara, CA, USA). After a wash, samples were analyzed with FACS Canto II flow cytometer (Becton Dickinson, New Jersey, USA). Mouse IgG1 (Biolegend) was used as isotype control to determine the non-specific binding of each marker.

### Chondrogenic and osteogenic differentiation capacity

The differentiation potential of both iliac crest and proximal tibia cells was evaluated in monolayers. BMAC from each sample was seeded onto a 12-well plate in α-MEM with 15% FBS. To assess chondrogenic differentiation, the standard culture medium was replaced after 24 h with a chondrogenic medium consisting of Dulbecco′s modified Eagle’s medium high glucose (DMEM, Sigma-Aldrich) with 10% FBS, 100X ITS-Premix (BD Biosciences, Bedford, MA), 10^–7^ dexamethasone (Sigma-Aldrich), 37.5 g/mL ascorbate-2 phosphate (Sigma–Aldrich), 1 mM of sodium pyruvate (Sigma-Aldrich), pen-streptomycin (100 U/mL 100 g/mL, Gibco), and 10 ng/mL of TGF-β_1_ (Miltenyi Biotec B.V. & Co. KG, Bergisch Gladbach, Germany). For osteogenic differentiation, after 24 h in a standard culture medium, cells were cultured in an osteogenic medium composed of α-MEM (Gibco) with 15% FBS, 10^–7^ M dexamethasone (Sigma-Aldrich), 75 µg/ml ascorbate-2 phosphate (Sigma–Aldrich), and 0.01 mM β-glycerolphosphate (Sigma-Aldrich). The cells grown DMEM high glucose with 10% FBS and in α-MEM with 15% FBS without the specific growth factors were used for chondrogenic and osteogenic control, respectively. Media were changed twice a week and the cells were evaluated at 28 days by histological staining and gene expression analyses.

### Histological staining and spectrophotometric quantification

Chondrogenic differentiation was evaluated by Alcian Blue staining at 28 days. Briefly, after a wash with PBS, cultured cells were fixed in 10% neutral buffered formalin for 30 min at room temperature (RT). Then, cells were incubated in a 3% acetic acid solution and stained with 1% Alcian Blue (Sigma–Aldrich) solution for 30 min. Stained cells were then washed in running tap water and rinsed in double deionizer water (DDW). To assess osteogenic differentiation, Ca^2+^ deposition was evaluated at 28 days by Alizarin Red S staining. Briefly, cells were washed with PBS and fixed in 10% neutral buffered formalin for 1 h at RT. After washes in DDW, cells were dehydrated and stained with 1% Alizarin red S (Sigma-Aldrich). To remove non-specific precipitation, cells were washed extensively with DDW.

To quantify the glycosaminoglycan production and the mineral deposition in both crest and tibial samples, a spectrophotometric analysis was performed using the TECAN Infinite^®^ 200 PRO device (Tecan Italia S.r.l., Italy). The absorption peaks were defined by analyzing a single scan of wavelength (230–1000 nm) of a small group of samples. The right wavelengths, 610 nm for Alcian Blue and 510 nm for alizarin red were selected and the analysis was performed reading the plates with the “multiple reads per well” method, which provided a mean value obtained from 177 fields per well, which allows the major level of accuracy provided by the instrument.

### Analysis of mRNAs expression by real-time PCR

Cells from both iliac crest and tibia were analyzed by real-time RT-PCR at 0 and 28 days to investigate the expression of specific markers during chondrogenic and osteogenic differentiation. The expression of collagen type II, SOX-9, and aggrecan was evaluated in chondrogenic cells, while alkaline phosphatase (ALP), osterix (OSX), and runt-related transcription factor-2 (RUNX-2) were chosen to assess the osteogenic pattern. RNA was isolated using TRIzol reagent (Invitrogen) following the manufacturer’s recommended protocol. After treatment with DNase I (DNA-free Kit; Ambion, Life Technologies), total RNA was quantified using a Nanodrop^®^ spectrophotometer (EuroClone S.p.a.). The RNA was reverse transcribed using MuLV reverse transcriptase (Thermo Fisher Scientific, Waltham, Massachusetts, USA). PCR primers for the selected genes and for the housekeeping gene glyceraldehyde-3-phosphate dehydrogenase (GAPDH) used as internal control are listed in Table 1. Real-time PCR was run in a LightCycler Instrument (Roche Molecular Biochemicals, Indianapolis, IN) using SYBR Premix Ex Taq (Takara, Clontech Laboratories, Mountain View, CA) with the following protocol: initial activation at 95 °C for 10 min, amplification for 45 cycles at 95 °C for 5 s and 60 °C for 20 s. mRNA levels were calculated for each target gene and normalized using the reference gene GAPDH according to the formula 2^−*Δ*Ct^.

**Table 1:** List of primers used in real-time PCR

RNA template	Primer sequences (5′-3′)	Annealing temperature (°C)
GAPDH	5′-TGGTATCGTGGAAGGACTCATGAC 3′-ATGCCAGTGAGCTTCCCGTTCAGC	60
ALP	5′-GGAAGACACTCTGACCGT 3′-GCCCATTGCCATACAGGA	60
RUNX-2	5′-GGAATGCCTCTGCTGTTATG 3′-AGACGGTTATGGTCAAGGTG	60
OSX	5′-TGCTTGAGGAGGAAGTTCACTATG 3′-AAAGGTCACTGCCCACAGA	60
SOX-9	5′-GAGCAGACGCACATCTC 3′-CCTGGGATTGCCCCGA	60
COLLAGEN TYPE II	5′-GACAATCTGGCTCCCAAC 3′-ACAGTCTTGCCCCACTTAC	60
AGGRECAN	5′-TCGAGGACAGCGAGGCC 3′-TCGAGGGTGTAGCGTGTAGAGA	60

### Statistical analysis

Data are presented as medians, quartiles, minimum and maximum values, percentages, and means ± standard deviations, as appropriate. The Levene test was performed to assess the homogeneity of variances. The Shapiro test was used to assess the normality of the distributions. Bivariate analysis: the paired *T* test was performed to assess the differences at different follow-up times and different sites (iliac crest, proximal tibia). When the sample size was less than 10, the paired *T* test was performed using the bootstrap method. The analysis of variance (ANOVA) was performed to assess the between-groups differences in continuous, normally distributed, and homoscedastic data; the Mann–Whitney *U* test (two groups) or the Kruskal Wallis test (more than 2 groups) was used otherwise. The generalized linear mixed models analysis with the studied parameters as dependent variables, follow-up times, site (iliac crest, proximal tibia) as fixed effects, and patient as random effect was used as multivariate analysis. The chosen distribution and link function was determined according to the distribution of the parameters: so far, we used gamma distribution with log-link function for strongly asymmetrical and non-normally distributed variables; Tweedie distribution with log-link function for asymmetrical and non-normally distributed variables with many 0 values; normal distribution with log-link function for asymmetrical log-normally distributed variables. With ten samples per group for both the comparisons of the number of mononuclear cells per ml between younger and older patients and between the iliac crest and proximal tibia sites of the older patients, and assuming an effect size equal to 1.54, a post hoc power equal to 0.9 was obtained. The level of statistical significance was set at *p* < 0.05. All statistical analysis was performed using SPSS v.19.0 (IBM Corp., Armonk, NY, USA).

## Results

### Mononucleated cell count and CFU-F assay

A total of 30 BMAC samples were analyzed, of which 10 were from younger patients and 20 from older patients: 10 from the anterior iliac crest and 10 from the tibia.

**Fig. 1:**
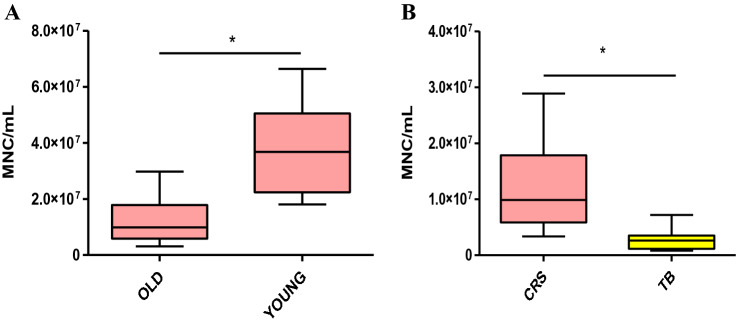
**A** Tukey box plots of the number of mononuclear cells (MNC)/mL obtained from “young” and “old” patients *P < 0.0005. **B** Tukey box plots of the number of mononuclear cells (MNC)/mL obtained from the iliac crest (CRS, pink) and proximal tibia (TB, yellow) samples. *p < 0.0005

The comparative analysis of BMAC characteristics based on patient age was performed on 10 iliac crest samples from younger patients and 10 iliac crest samples from older patients. A significantly higher number of mononuclear cells per ml were observed in younger patients (3.8 ± 1.8 × 10^7^) compared to older patients (1.2 ± 0.8 × 10^7^) (*p* < 0.0005) (Fig. 1A).

**Fig. 2:**
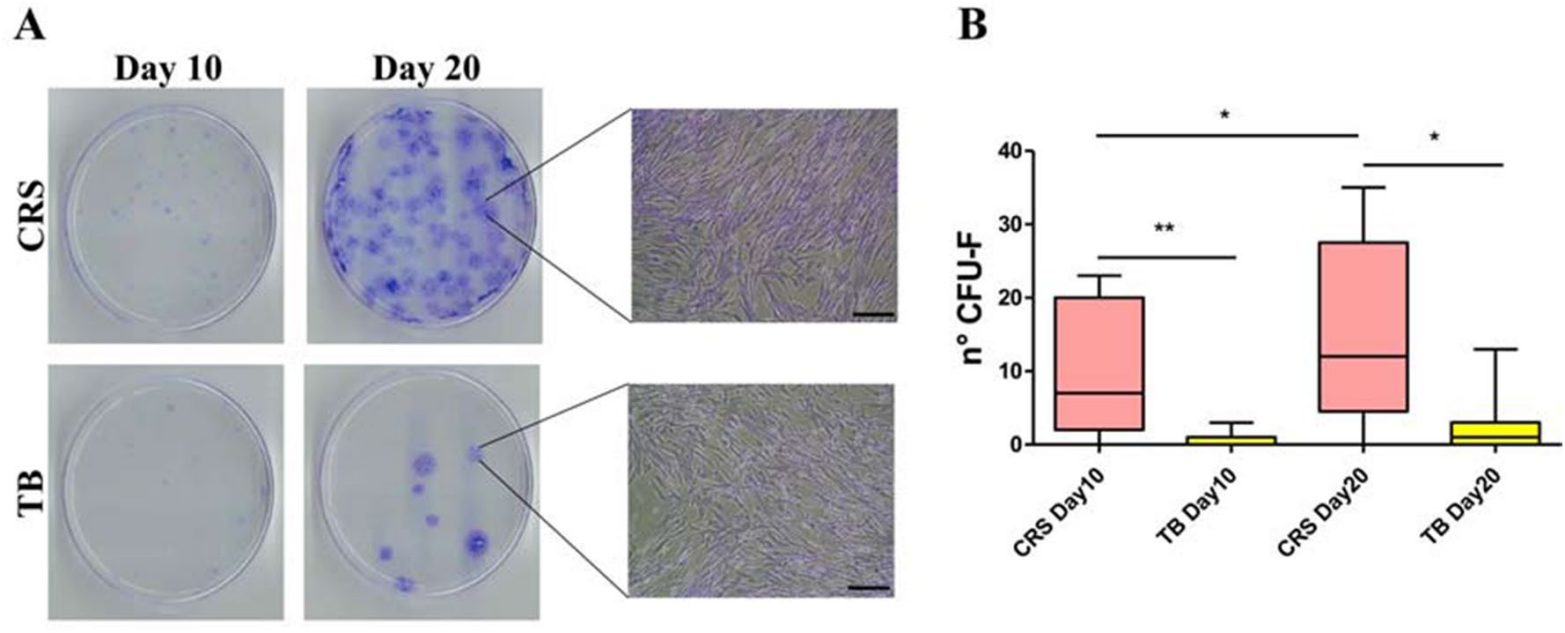
**A** Representative images of CFU-F and inverted microscopy of cells from donor-matched BMAC samples (older patient group) harvested from the iliac crest (CRS, pink) and proximal tibia (TB, yellow) on days 10 and 20; scale bar: 100 μm. **B** Tukey box plots show the number of CFU-F from CRS and TB cells on days 10 and 20. *p < 0.05, **p < 0.005

The possible influence of the harvest site on the BMAC characteristics was evaluated by comparing ten donor-matched samples from the iliac crest with ten from the proximal tibia (older group). The mean number of mononuclear cells per ml of BMAC was significantly higher in the samples harvested from the iliac crest than from the proximal tibia (1.2 ± 0.8 × 10^7^ vs 0.3 ± 0.2 × 10^7^, respectively, *p* < 0.0005) (Fig. 1B). This result was confirmed by the CFU-F analysis: the iliac crest BMAC samples showed a significantly higher number of CFU-F compared to the tibial BMAC samples both at day 10 (15.9 ± 19.4 vs 0.6 ± 1.0, *p* = 0.001) and at day 20 (21.7 ± 23.0 vs 2.9 ± 4.2, *p* = 0.006) (Fig. 2). The iliac crest BMAC showed a significant increase in the number of CFU-F from day 10 to day 20 (*p* = 0.020), while the tibial BMAC showed only a tendency (*p* = 0.063).

### Phenotype characterization of BMSCs from the iliac crest and proximal tibia

**Fig. 3:**
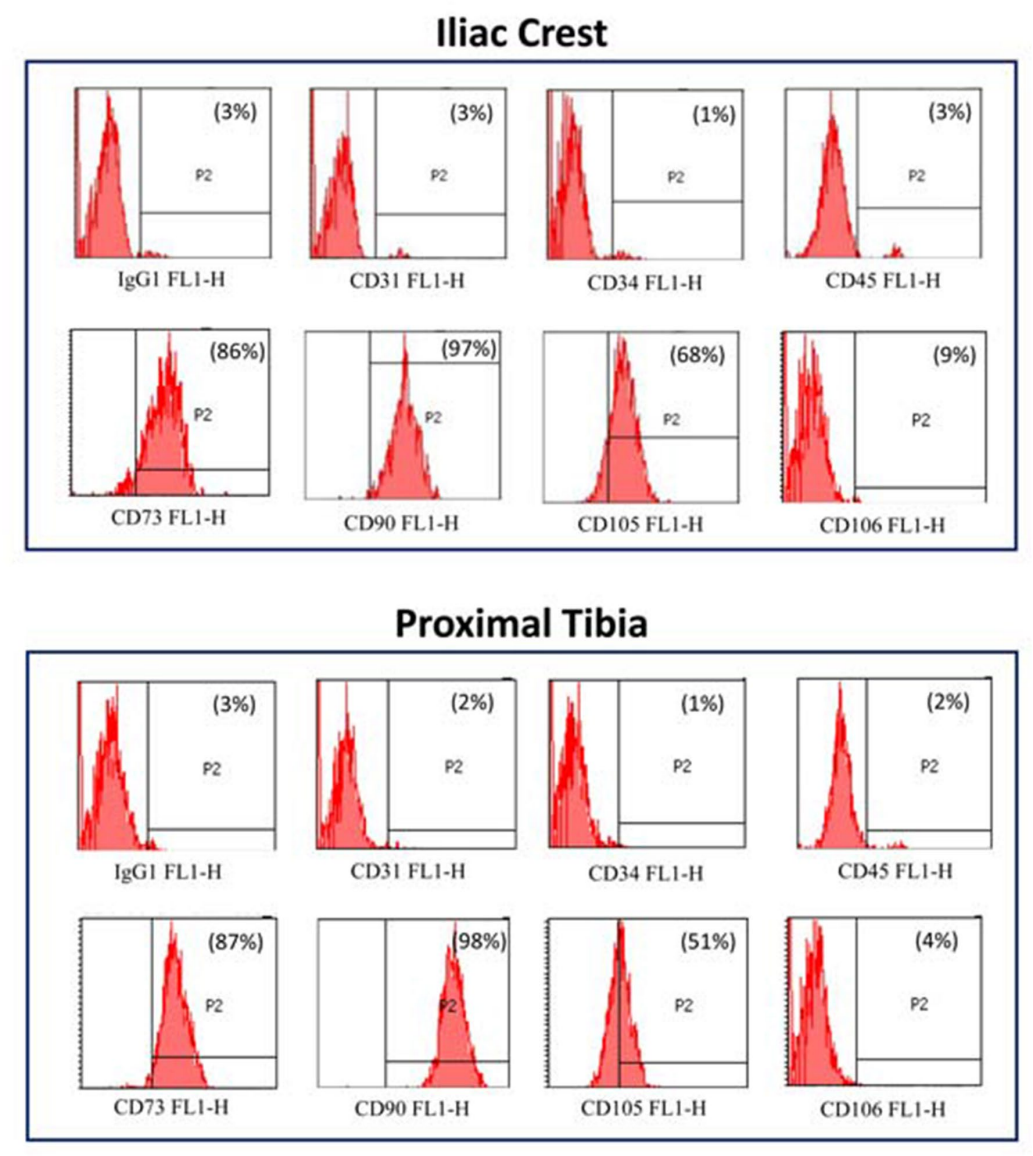
FACS analysis of typical hematopoietic and mesenchymal markers in a representative sample of BMSC, from both crest and proximal tibia. Histograms reporting the percentage of positivity (P2) of the following markers: CD-31, CD-34, CD-45, CD-73, CD-90, CD-105, CD-106, and the relative isotype controls

Both cells derived from iliac crest and proximal tibia showed the same phenotypic pattern at flow cytometry. The two harvest sites revealed a comparable percentage of expression for all the markers evaluated, with a positive expression for the mesenchymal markers CD73, CD90, CD105, and CD106, and a negative expression for the hematopoietic markers CD34 and CD45, and for CD31 (Fig. 3). Only the CD105 marker was more expressed in BMSCs from the iliac crest compared to the proximal tibia (*p* = 0.041).

### Chondrogenic and osteogenic potential of BMAC from iliac crest and proximal tibia

For both chondrogenic and osteogenic differentiation assay, the cells grown for 28 days in non-inducing media (CTR) did not proliferate sufficiently to extract their RNA. Thus, CTR was excluded from the statistical analysis of molecular biology evaluation.

**Fig. 4:**
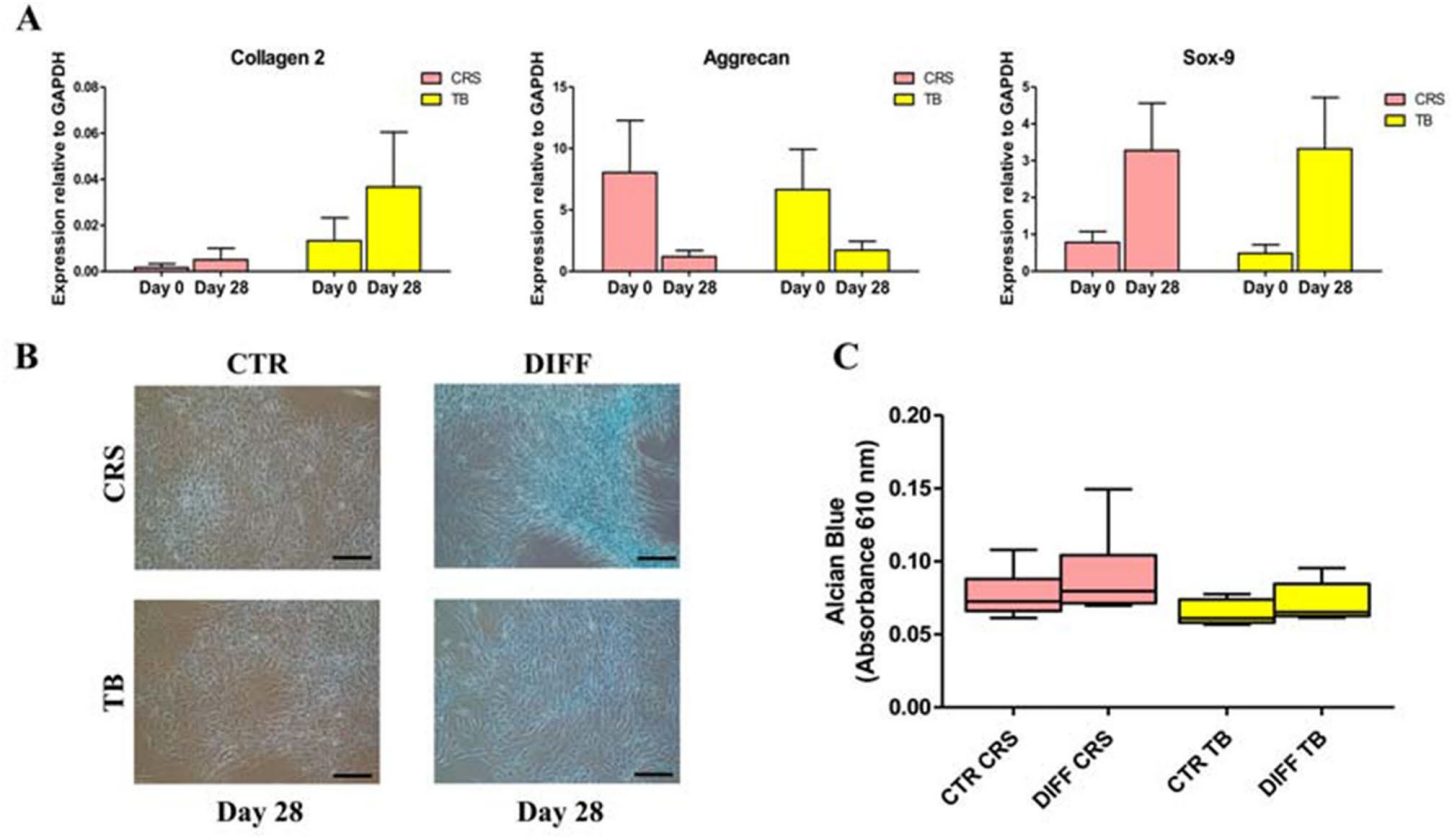
Chondrogenic differentiation potential. **A** Gene expression of cells derived from BMAC samples (older patient group) harvested from the iliac crest (CRS, pink) and proximal tibia (TB, yellow) on day 0 and after 28 days in chondrogenic medium (DIFF). **B** Representative images of cells stained with Alcian Blue on day 28 in control (CTR) and differentiated (DIFF) cells. Scale bar: 100 μm. **C** The panel shows the chondrogenic cell differentiation on day 28 reported as absorbance values at 610 nm in CRS (CRS, pink) and TB (TB, yellow) samples

The chondrogenic potential was evaluated for the iliac crest and proximal tibia donor-matched samples derived from six patients of the older group, since in four patients the cells did not grow sufficiently to perform the analyses. The cells derived from the iliac crest and proximal tibia showed a similar trend for all the genes evaluated. No statistically significant modulation in terms of mRNA levels of collagen type II, SOX-9, and aggrecan were observed from day 0 to day 28 in chondrogenic medium (DIFF), as well as no significant differences were observed between the two groups (iliac crest vs proximal tibia) (Fig. 4A). This result was confirmed by Alcian Blue staining, which displayed the presence of glycosaminoglycans on day 28 only for DIFF samples compared to CTR ones with no significant difference between iliac crest- and tibia-derived BMSCs (Fig. 4B, C).

**Fig. 5:**
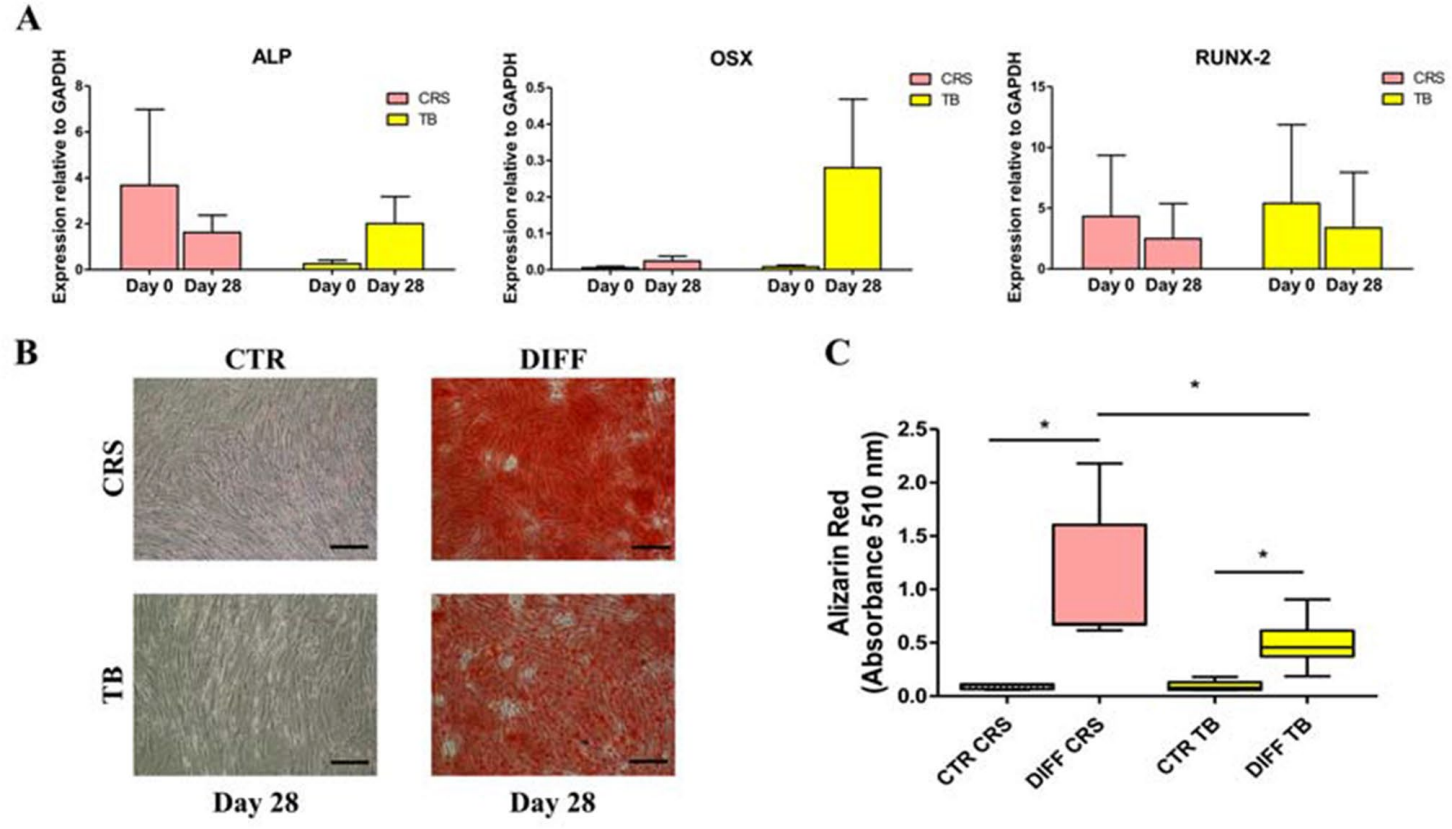
Osteogenic differentiation potential. **A** Gene expression of cells derived from BMAC samples (older patient group) harvested from iliac crest (CRS, pink) and proximal tibia (TB, yellow) on day 0 and after 28 days in osteogenic medium (DIFF). **B** Representative images of cells stained with Alizarin Red on day 28 in control (CTR) and differentiated (DIFF) cells. Scale bar: 100 μm. **C** Chondrogenic cell differentiation on day 28 reported as absorbance values at 510 nm in CRS (CRS, pink) and TB (TB, yellow) samples. *p < 0.05

The osteogenic differentiation potential was evaluated for iliac crest and proximal tibia donor-matched samples derived from seven patients, since for the remaining three patients the cells did not grow sufficiently to perform the analyses. The cells from the iliac crest and proximal tibia showed comparable osteogenic differentiation potential. Regarding gene expression, osteogenic induction promoted a weak production of ALP, OSX, and RUNX-2 with no significant differences from day 0 up to 28 days in both groups (Fig. 5A). In contrast, histological evaluation showed a stronger mineral production on day 28 in DIFF samples with respect to CTR (*p* = 0.016) in both iliac crest and proximal tibia samples, with a higher Ca^2+^ apposition in iliac crest-derived BMSCs compared to the tibia (*p* = 0.006) (Fig. 5B, C).

## Discussion

The main finding of this study was that harvest site and patient age significantly influence BMAC cell features. While BMSCs from BMAC harvested from iliac crest and proximal tibia showed a similar expression of mesenchymal markers and similar osteogenic and chondrogenic differentiation potential, BMAC from the iliac crest displayed a significantly higher number of mononucleated cells and CFU-F compared to tibial BMAC harvested from the same patients. Moreover, a significantly higher number of mononucleated cells were found in BMAC samples from the iliac crest of younger patients, showing an age-related influence on BMAC composition.

The rationale for the use of BMAC relies on the transplantation of the entire bone marrow “niche” containing BMSCs, hematopoietic precursors, monocytes, platelets, endothelial cells, as well as a great array of soluble factors [23, 25]. The combination of cells and bioactive molecules can promote tissue healing and improve clinical results in different pathological conditions [25]; therefore, BMAC has been widely used for the treatment of a variety of musculoskeletal pathologies, from bony defects and tendon injuries to cartilage defects and OA [9, 18, 26, 27, 38, 45, 59].

Nevertheless, clear evidence on its mechanism of action in different scenarios is still lacking. Several preclinical and clinical studies described a direct relationship between an increased therapeutic effect and the number of progenitor cells in BMAC [11, 29, 31, 61], which supports efforts toward maximizing BMSC content to fully exploit the therapeutic potential of this biological product. To standardize and optimize the use of BMAC in orthopedic clinical practice, several studies have been performed to investigate possible influencing factors [5, 35]. The bone marrow harvesting procedure is important in determining the number of BMSCs contained in BMAC. In particular, the collection of larger bone marrow aspirate volumes with high volume syringes seem to decrease the concentration of BMSCs because of the dilution of the bone marrow sample with peripheral blood [49, 49]. The needle used for bone marrow harvesting could be another factor affecting the quality of BMAC and its amount of BMSCs [21, 40, 42, 58]. Some authors also suggested that a multiple-site harvest can be superior versus a single-site harvest [51]. Besides the factors related to the harvest technique, the biological properties of BMAC also appear to be strongly related to the harvest site.

BMAC is typically isolated from the iliac crest (anterior or posterior), which is considered the “gold standard” due to its prominence and ease of collection [47]. Nevertheless, there is a recent increase of interest in an even simpler approach to retrieve BMAC by harvesting bone marrow directly from the proximal tibia instead of the iliac crest when treating knee pathologies. In fact, this would shorten and simplify the overall procedure by addressing the same joint while avoiding the risk of adverse events in other locations like the iliac crest. However, only a limited number of studies compared BMSC amounts from different bone marrow harvest sites. Pierini et al. analyzed the number of cells, concentration, and prevalence of CFU-F obtained from the anterior and posterior iliac crest. A higher quality of bone marrow aspirate was observed when it was collected from the posterior, rather than anterior iliac crest, with a 1.6 times greater yield of CFU-F in posterior iliac crest specimens [52]. Hyer et al. compared the yield of BMSCs obtained from bone marrow aspirate of the anterior iliac crest with that obtained from the distal tibial metaphysis and calcaneal body, two alternative harvest sites often used for ankle surgery. The authors reported that, while all tested bone marrow sites contained progenitor cells, the iliac crest provided the greatest yield of BMSCs [32]. McLain et al. evaluated the quality of bone marrow aspirate harvested from vertebral bodies and demonstrated that biologic activity and prevalence of the connective tissue progenitor cells were comparable with those of cells from the iliac crest, suggesting that this alternative marrow source may further reduce the time and morbidity associated with iliac crest harvest during spinal surgery [48]. Two studies compared samples from iliac crest with proximal tibia and distal femur in old patients who underwent total knee arthroplasty [16, 50]. Both studies suggested that the iliac crest was superior to femur and tibia in terms of the number of BMSCs isolated, although there was no significant difference in the phenotype of the cells isolated from different locations, with common BMSC surface markers and comparable differentiation capacity of the cells isolated from different locations.

Considering the constant increase in the elderly population, OA is and will be a growing clinical need to be addressed. In the attempt to postpone joint replacement, several conservative options have been proposed, among which the use of orthobiologics seems promising [22]. In this view, this in vitro study was conducted on BMAC samples derived from the iliac crest and proximal tibia of the same patients, with a mean age of 57 years and mild OA level. The flow cytometer analysis did not show significant differences in the expression of mesenchymal markers between the iliac crest and tibial samples, except for CD105, a marker of MSC phenotype, which was more expressed in cells derived from the iliac crest compared to the proximal tibia. Several studies suggested a relation between CD105 expression and the differentiation potential of MSCs. CD105-positive MSCs, showed a better chondrogenic differentiation compared to CD105-negative cells, which were more osteogenic [34, 36, 41]. On the other hand, Cleary et al. [13] demonstrated that CD105 expression was not related to MSC chondrogenic capacity, confirming our findings, in which no differences were observed in terms of chondrogenic potential in cells obtained from both iliac crest and proximal tibia. Moreover, BMAC obtained from the two harvest sites also demonstrated the same osteogenic potential in terms of gene expression, even if an increase in the mineral matrix apposition in iliac crest cells was evident. This high mineralization could be due to the presence, in bone marrow from the iliac crest, of more cells with high differentiation potential. Moreover, it should be considered that in an in vitro system, gene expression changes over time, and it is not directly correlated with matrix protein production. The high CD105 expression is also associated with a great CFU-F number in iliac crest cells compared to the proximal tibia ones. These findings were confirmed by Kastrinaki et al. [37], who demonstrated a correlation between CFU-F and CD105 cell expression, suggesting, furthermore, that the CD105 cell fraction contains an immature MSC population.

Overall, these data indicate that both iliac crest and proximal tibia contain BMSCs with comparable multipotent characteristics, but iliac crest possesses a clearly higher frequency of BMSCs as demonstrated by its higher number of mononucleated cells and CFU-F compared to the proximal tibia of the same patients. In particular, BMAC from the iliac crest displayed a four times higher number of mononucleated cells compared to the proximal tibia, as well as a higher clonogenic capacity. This should be considered when choosing the harvest site to obtain BMAC, especially in a one-step procedure where cells are non-expanded.

This study also showed that patient age affects BMAC composition; a three times higher number of mononucleated cells were found in BMAC from the iliac crest of younger patients compared to older patients. This confirms the results of previous reports demonstrating that aging correlated with the depletion of the available stem cell pool in the bone marrow [1]. Some authors also reported changes in terms of BMSC quality in BMAC depending on age, with an age-related reduction of the proliferation and differentiation capacity [2, 44, 57]. Based on these results, the quality of BMSCs seems higher in young patients, suggesting that a reduced biologic potential using BMAC in older patients could be expected. Still, the BMAC approach provided satisfactory results in some clinical trials on adult populations [4, 9, 38]. It would be important to understand if the benefits observed in these studies depend mainly on a placebo effect, which was suggested to play an important role in innovative treatments such as orthobiologic products [53], or if the quality of BMAC in older patients is still sufficient to provide significant clinical improvements. In fact, it has to be considered that the mechanism of action of these POC products does not only rely on the frequency of cells and their differentiation capacity, but also on their interactions with resident cells by paracrine action, especially when administered by intra-articular injections.

Also, BMAC efficacy seems not to solely depend on the presence of BMSCs as their frequency in the bone marrow is extremely low, ranging from 1/10,000 to 1/100,000 of total nucleated cells [17]. BMAC benefits could also be ascribed to the high concentrations of platelets and bioactive molecules [39]. BMAC contains a high concentration of growth factors which are reported to have anabolic and anti-inflammatory effects [33, 46, 56]. Among these, it has been reported that BMAC has a considerable concentration of interleukin-1 receptor antagonist (IL-1ra), which inhibits IL-1 catabolism and therefore may be responsible for the beneficial symptomatic pain relief with this biological approach [8, 12, 48]. Future studies should investigate the role of the “niche”, platelets, and growth factors present in BMAC both in preclinical and clinical studies to better understand their contribution to BMAC efficacy, focusing on the factors released by cells in response to the pathological environment. In this perspective, further evaluation of the so-called “secretome” (all the factors secreted by the cell, along with the constituents of the secretory pathway) and in particular of the active therapeutic agents herein contained, such as microvesicles and exosomes, will be able to clarify the mechanism of action of BMAC and other orthobiologics [14, 15, 54].

This study presents some limitations. First, the number of patients analyzed did not allow further sub-analyses. Nevertheless, the number of samples evaluated is in line with previous similar studies on this topic. Moreover, considering the high level of inter-donor variability in BMSC amount, which limits the power of the conclusions of previous studies, the use of donor-matched comparisons is a strength of this study that increases the value of the results obtained. Second, for some samples, it was not possible to obtain enough cells to carry on all the analyses planned, mainly due to the poor proliferative capacity of some of the cell batches, in particular for those obtained from the proximal tibia, which, indeed, can be considered a result per se. Third, while 3D pellet culture models are often used to investigate the chondrogenic differentiation, in the present experiments pellet cultures could not be obtained by seeding directly BMAC, and therefore a monolayer approach was used as previously reported [10]. Moreover, due to ethical reasons, it was only possible to have samples from the iliac crest and proximal tibia in the older populations, since the samples were based on materials remaining from surgical procedures planned for therapeutic aims, which were not available in the procedures for the young population. Finally, the prevalence of men compared to women did not allow analyzing sex influence on BMAC quality. Therefore, future studies should analyze BMAC quality in larger populations to investigate the factors that may influence and optimize BMAC quality and its potential in terms of clinical effects.

## Conclusions

Harvest sites and age can affect the properties of BMAC in terms of cell number, clonogenic capacity, and differentiation potential. BMSCs obtained from the iliac crest and proximal tibia of the same patients present a similar mesenchymal marker expression as well as osteogenic and chondrogenic differentiation capacity. However, iliac crest BMAC presents a four times higher number of mononucleated cells with significantly higher clonogenic capacity compared to the tibia. Age also influences BMAC quality, with a three times higher number of mononucleated cells in younger patients. The identification of BMAC characteristics could help to optimize its preparation and identify the most suitable indications for this orthobiologic treatment in clinical practice.
